# Efficacy and safety of tocilizumab in Chinese patients with systemic juvenile idiopathic arthritis: a multicentre phase IV trial

**DOI:** 10.1007/s10067-024-07126-9

**Published:** 2024-09-16

**Authors:** Caifeng Li, Xuemei Tang, Zhixuan Zhou, Li Sun, Meiping Lu, Wei Zhou, Sirui Yang, Wenjie Zheng, Haiguo Yu, Weiping Tan, Junmei Zhang, Yu Zhang, Yuxiu Kong, Jiahui Xu

**Affiliations:** 1 Department of Rheumatology, Beijing Children’s Hospital, Capital Medical University, National Center for Children’s Health, No. 56 South Lishi Road, Xicheng District, Beijing, 100045 China; 2 Department of Rheumatology and Immunology, Children’s Hospital of Chongqing Medical University, Chongqing, China; 3 Department of Rheumatology and Immunology, Children’s Hospital Capital Institute of Pediatrics, Beijing, China; 4 Department of Rheumatology, Children’s Hospital of Fudan University, Shanghai, China; 5 Department of Rheumatology, Immunology and Allergy, Children’s Hospital Affiliated to Zhejiang University School of Medicine, Zhejiang, China; 6 Department of Nephrology, Shanghai Children’s Medical Center Affiliated to Shanghai Jiaotong University School of Medicine, Shanghai, China; 7 Department of Pediatric Rheumatology and Allergy, The First Hospital of Jilin University, Jilin, China; 8 Department of Pediatric Rheumatology, The Second Affiliated Hospital and Yuying Children’s Hospital of Wenzhou Medical University, Zhejiang, China; 9 Department of Rheumatology and Immunology, Children’s Hospital of Nanjing Medical University, Jiangsu, China; 10 Department of Pediatrics, Sun Yat-Sen Memorial Hospital, Sun Yat-Sen University, Guangdong, China; 11 Medical Affairs, Shanghai Roche Pharmaceuticals Co., Ltd., Shanghai, China

**Keywords:** Biological therapy, Systemic juvenile idiopathic arthritis, Tocilizumab

## Abstract

**Objectives:**

Given the limited tocilizumab (TCZ) treatment data for systemic juvenile idiopathic arthritis (sJIA) in China, we evaluated the long-term efficacy and safety of TCZ in Chinese patients with sJIA.

**Method:**

In this multicentre, interventional Phase IV study, patients with sJIA and inadequate clinical response to non-steroidal anti-inflammatory drugs/corticosteroids received TCZ infusions every 2 weeks based on body weight (< 30 kg, 12 mg/kg; ≥ 30 kg, 8 mg/kg), over a 52-week open-label period and an 8-week safety follow-up period. The primary endpoint was the proportion of patients with a JIA American College of Rheumatology (ACR) 30 response and absence of fever at Week 12.

**Results:**

Sixty-two patients were enrolled and treated (12-mg/kg group, 34; 8-mg/kg group, 28). At Week 12, 87.1% (95% confidence interval 78.8%–95.4%) of patients had JIA ACR 30 response and absence of fever; Week 52 results were similar. The proportion of JIA ACR 30/50/70/90 responders rapidly increased at Week 12, up to Week 52. High-sensitivity C-reactive protein (hsCRP) levels decreased within 4 weeks; 44/58 patients (75.9%) with elevated baseline hsCRP recovered at Week 52. Childhood Health Assessment Questionnaire pain scores, disability index scores, and mean corticosteroid dose decreased over time. Height standard deviation score changes at Week 52 indicated catch-up growth. Most adverse events (AEs) were mild (serious AE incidence, 17.7%). No deaths or macrophage activation syndrome occurred.

**Conclusion:**

This is the first multicentre trial to report the efficacy and safety of TCZ in Chinese patients with sJIA at 52 weeks. No new safety concerns were found.

**Supplementary Information:**

The online version contains supplementary material available at 10.1007/s10067-024-07126-9.

**Table:** 

**Key points** *• This is the first multicentre trial providing strong evidence for tocilizumab (TCZ) treatment for systemic juvenile idiopathic arthritis (sJIA) in China.* *• The study reported TCZ had good efficacy and favourable safety profiles in Chinese sJIA patients in the long term (52 weeks).* *• TCZ treatment showed rapid disease control, which was maintained over time, catch-up growth benefits in patients, tapering and discontinuation of corticosteroids, and improved quality of life.*		

## Introduction

Systemic juvenile idiopathic arthritis (sJIA) is a rare paediatric autoinflammatory disease and a distinctive subtype of juvenile idiopathic arthritis [1, 2]. The estimated global prevalence of sJIA ranges from 3.8 to 400/100,000 [3]. While sJIA accounts for 5% to 15% of all JIA cases in North America and Europe, in Asia, it may account for a greater proportion [4].

The early clinical manifestations of sJIA are characterised by autoinflammatory features such as spiking fever, erythematous rash, and elevated inflammatory markers; arthritis may or may not be present in the early stages of the disease [1, 2, 5]. The disease course can vary at later stages. The diagnosis of sJIA requires the presence of arthritis and fever within the last 2 weeks as well as one of the following criteria: rash, generalised lymphadenopathy, enlargement of liver or spleen, or serositis [6]. Here, laboratory tests (e.g., elevated erythrocyte sedimentation rate [ESR], C-reactive protein) may be useful to define disease activity. A study found that 51%, 42%, and 7% of patients with sJIA had a persistent, monocyclic, and polycyclic disease course, respectively (mean follow-up: 5 years) [7]. The presence of hepatosplenomegaly, lymphadenopathy, and/or serositis separates sJIA from other subtypes of JIA [5]. sJIA affects both male and female patients, although the risk has been reported to be higher in girls [3]. Some complications of sJIA include stunted growth and macrophage activation syndrome (MAS), which is characterised by persistent fever, cytopenias, liver abnormalities, coagulopathy, and central nervous system dysfunction and can be life-threatening [8, 9].

Conventional treatment options for sJIA include non-steroidal anti-inflammatory drugs (NSAIDs), corticosteroids, and conventional synthetic disease-modifying anti-rheumatic drugs. Early treatment of sJIA is important to prevent joint damage and disability and to control systemic symptoms. However, achieving adequate disease control remains challenging in some patients because of limited treatment efficacy. Furthermore, long-term use of corticosteroids can cause a wide range of side effects.

Tocilizumab (TCZ) is a recombinant humanised, anti-human monoclonal antibody of the immunoglobulin subclass directed against soluble and membrane-bound interleukin-6 (IL-6) receptors. IL-6 is a pro-inflammatory cytokine that plays a significant role in the pathogenesis of sJIA [10, 11]. IL-6 binds to the IL-6 receptor, forming a hexameric receptor complex that activates intracellular signalling pathways involving Janus kinase and signal transducer and activator of transcription pathways, leading to local and systemic inflammation [12]. Increased production of IL-6 is associated with thrombocytosis, microcytic anaemia, growth retardation, and osteopenia observed in patients with sJIA, and serum IL-6 levels increase/decrease in parallel with the fever spikes [13].

TCZ is approved for the treatment of sJIA in the United States, Europe, and other countries, and was approved for the treatment of sJIA in China in October 2016 based on the results of a global, 12-week, randomised, double-blinded, placebo-controlled, Phase III study [14]. TCZ has been recommended as a first-line biological agent in the 2021 American College of Rheumatology (ACR) guidelines [15]. However, there is currently limited data on the efficacy and safety of TCZ in Chinese patients with sJIA, especially in the long term. Therefore, this is first study to evaluate the long-term (52 week) efficacy and safety of treatment with TCZ in Chinese patients with sJIA.

## Methods

### Study design

This was a single-arm, open-label, Phase IV study conducted at 10 centres in China. A list of the study sites is included in the Supplementary Methods. The study had a 3-week screening period, a 52-week open-label period with patient visits every 2 weeks, and an 8-week safety follow-up period. Data were collected using electronic case report forms with each study site entering their data into an electronic data capture system.

The study protocol was approved by the Institutional Review Board/Independent Ethics Committee at each centre, and the study was conducted in accordance with the Declaration of Helsinki, International Ethical Guidelines of the Council for International Organizations of Medical Sciences, and International Conference on Harmonisation Guideline for Good Clinical Practice. All patients or their legal guardians provided written informed consent. This study was registered at ClinicalTrials.gov (NCT03301883).

### Patients and treatment

The main inclusion criteria were as follows: Chinese patients aged 2–17 years at the screening visit, meeting the International League of Associations for Rheumatology classification for sJIA, > 6 months of documented persistent sJIA activity prior to screening, including an inadequate response to NSAIDs and corticosteroids due to toxicity or lack of efficacy, with active disease (defined as ≥ 5 active joints at screening and baseline or ≥ 2 active joints at screening and baseline with temperature > 38˚C for ≥ 5 out of any 14 consecutive days during screening). The main exclusion criteria were: patients with any other autoimmune, rheumatic disease or syndrome other than sJIA; any significant concurrent medical or surgical condition that would jeopardise the patient’s safety or ability to complete the trial; evidence of serious uncontrolled concomitant diseases, including, but not limited to, the nervous system, renal, hepatic, or endocrine diseases.

Patients received a TCZ infusion every 2 weeks, with the dosing regimen based on their body weight as follows: 12 mg/kg for patients with body weight < 30 kg or 8 mg/kg for patients with body weight ≥ 30 kg. NSAIDs, methotrexate (MTX), and oral corticosteroids were permitted during the study, but not required. Among patients undergoing treatment with NSAIDs, MTX, and oral corticosteroids, the dose of NSAIDs and MTX remained stable during the first 12 weeks of the study, and the corticosteroid dose remained stable during the first 6 weeks of the study, after which the doses could be adjusted as needed. If an intra-articular injection was necessary, no more than one joint was allowed to be injected with the lowest possible dose relative to the size of the joint. Disease-modifying anti-rheumatic drugs other than MTX and other biologics were not allowed.

### Endpoints

#### Primary efficacy endpoint

To assess the efficacy of TCZ in combination with stable ongoing therapy regarding signs and symptoms in patients with sJIA with persistent activity and an inadequate response to NSAIDs and systemic corticosteroids, the proportion of patients with a JIA ACR30 response and absence of fever at Week 12 was evaluated as the primary efficacy endpoint.

The six JIA ACR core components consist of physician global assessment of disease activity and parent/patient global assessment of overall well-being (both 100-mm Visual Analogue Scales), number of joints with active arthritis, number of joints with limitation of movement, ESR, and functional ability (Childhood Health Assessment Questionnaire [CHAQ]). At an assessment visit, a JIA ACR30 response was defined as at least three of the six JIA ACR core components improving by ≥ 30% from the baseline assessments and no more than one of the remaining JIA ACR core components worsening by > 30% from the baseline assessments. For each JIA ACR core component, a ≥ 30% improvement was defined as a percentage change from baseline (CFB) ≤  − 30.

The absence of fever was defined as no temperature measurements ≥ 37.5˚C recorded (in the patient diary) in the 7 days preceding the day on which the JIA ACR core components were assessed.

#### Secondary efficacy endpoints

To assess the durability and extent of the TCZ efficacy response in patients with sJIA, the following endpoints were evaluated: proportion of patients with a JIA ACR30 response and absence of fever at Week 52; proportion of patients with a 30%, 50%, 70%, and 90% improvement in the JIA Core Set parameters at Weeks 12, 24, and 52; proportion of patients with inactive disease at Weeks 24 and 52; proportion of patients with clinical remission at Week 52; proportion of patients with an elevated (> upper limit of normal [ULN]) high-sensitivity C-reactive protein (hsCRP) at baseline who had normal (≤ ULN) hsCRP at Weeks 12, 24, and 52; and mean value and mean CFB in the CHAQ pain score (range, 0–100) assessed over time.

To assess the efficacy of treatment with TCZ to permit concomitant medication reduction or elimination, the following endpoints were evaluated: mean value and mean CFB in corticosteroids and/or MTX dose assessed over time and the proportion of patients who discontinued permitted concomitant medication for sJIA assessed over time.

#### Exploratory endpoints

To assess the effect of treatment on quality of life using the CHAQ and Child Health Questionnaire-Parent Form 50 (CHQ-PF50), the following exploratory endpoints were evaluated: proportion of patients with improvement compared with baseline in the CHAQ disability index assessed over time (improvement was defined as a decrease of at least 0.13 in the CHAQ disability index from baseline) [16], mean value and mean percentage CFB in the CHAQ disability index assessed over time, and mean value and mean CFB in the CHQ physical and psychosocial summary measures assessed over time.

To assess the effect of treatment on a patient’s growth, the mean height standard deviation score (SDS), as based on the appropriate Tanner stage, was assessed over time. In addition, the proportion of patients with a minimal clinical improvement (growth rate > 0.25 SDS) in SDS from baseline by Tanner stage grouping was also assessed.

Additionally, indicators of inflammation such as haemoglobin levels, platelet counts, and leukocyte counts were also analysed.

#### Safety endpoints

Adverse events (AEs), serious adverse events (SAEs), vital signs over time, and clinical laboratory tests over time were evaluated.

### Statistical methods

The target sample size was calculated based on the result of the previous global pivotal Phase III study, which reported that 85.3% of patients treated with TCZ had a JIA ACR30 response and absence of fever at Week 12 [14]. Thus, a sample size of 62 patients would provide an estimated precision of 10% for an observed proportion of patients with JIA ACR30 response and absence of fever at Week 12, which was 80% (95% confidence interval [CI], 70%–90%). Considering a drop-out rate of 5%, the target sample size was set at 65 patients. This sample size would also provide a > 95% probability to observe an AE incidence rate of ≥ 5%.

The intention-to-treat (ITT) population was defined as all patients who were enrolled and who received at least one dose of the study drug. The safety population, defined as all patients who were enrolled, received at least one dose of the study drug, and had at least one post-enrolment safety assessment, was used for the analysis of safety endpoints. Other analyses were based on the ITT population.

AEs and medical history were coded using MedDRA 25.0. AEs were graded by National Cancer Institute Common Terminology Criteria for Adverse Events (CTCAE) Version 4.03. Prior/concomitant/permitted concomitant medications were coded using WHODrug, version Global B3 March 2022.

In the primary analyses of assessing JIA ACR 30/50/70/90 responses, patients who withdrew before Week 12, 24, or 52 or for whom all six JIA ACR core components at Week 12, 24, or 52 could not be determined, including those who missed study visits because of travel restrictions due to the COVID-19 outbreak, were classified as JIA ACR 30/50/70/90 non-responders (non-responder imputation [NRI]). Sensitivity analyses were performed in patients that were observed at certain time points (observed).

Because there was no formal hypothesis testing for this single-arm study, descriptive statistics were used to report the results. All statistical analyses were performed using SAS software version 9.4 (SAS Institute Inc., Cary, NC, USA).

## Results

### Patients

Patients were recruited from 20 April 2018 to 4 June 2021, with the last patient visit on 14 June 2022. Sixty-four patients were screened, among whom two patients had screening failure as they did not meet the inclusion criteria of active disease; therefore, 62 were included in the ITT and safety populations (TCZ 12-mg/kg group [< 30 kg], n = 34; TCZ 8-mg/kg group [≥ 30 kg], n = 28). Fifty-six patients completed the study. Six patients discontinued from the study because of AEs (n = 4), physician decision (n = 1), and withdrawal by patient or legal guardian (n = 1) (Fig S1).

At baseline, the median (range) age of patients was 9.0 (2.0–16.0) years, and 46.8% were female. The median (range) duration of sJIA was 19.5 (1.0–84.0) months. The mean ± standard deviation (SD) hsCRP level was 59.2 ± 49.41 mg/L. Fifty-seven (91.9%) patients had background oral corticosteroid use, 53 (85.5%) patients had background MTX use, and 58 (93.5%) patients had background NSAIDs use. The mean ± SD sJIA ACR core components of patients at baseline were: physician’s global assessment of disease activity, 54.6 ± 20.95; parent’s/patient’s global assessment of overall well-being, 56.7 ± 28.60; number of joints with active arthritis, 7.7 ± 5.14; number of joints with limitation of movement, 5.1 ± 4.20; ESR, 61.0 ± 33.82 mm/h; and CHAQ disability index, 0.7 ± 0.73 (Table 1).

**Table 1:** Demographics and baseline clinical characteristics (ITT population)

	**TCZ overall population** **N = 62**	**TCZ 12-mg/kg group** **n = 34**	**TCZ 8-mg/kg group** **n = 28**
Sex, female, n (%)	29 (46.8)	15 (44.1)	14 (50.0)
Race, n (%)			
Han	59 (95.2)	32 (94.1)	27 (96.4)
Other	3 (4.8)	2 (5.9)	1 (3.6)
Age, years	9.0 (2.0–16.0)	6.5 (2.0–12.0)	12.0 (8.0–16.0)
Weight, kg	26.5 (11.5–75.0)	21.0 (11.5–29.2)	41.8 (30.0–75.0)
Body mass index, kg/m^2^	16.9 (12.9–33.8)	15.5 (12.9–21.0)	19.4 (15.5–33.8)
Duration of disease, months	19.5 (1.0–84.0)	16.5 (1.0–82.0)	25.5 (2.0–84.0)
Prior use of methotrexate, n (%)	53 (85.5)	25 (73.5)	28 (100.0)
Prior use of oral corticosteroids, n (%)	57 (91.9)	32 (94.1)	25 (89.3)
Prior use of NSAIDs, n (%)	58 (93.5)	31 (91.2)	27 (96.4)
Physician’s global assessment of disease activity mean ± SD	50.5 (5.0–100.0) 54.6 ± 20.95	53.0 (5.0–90.0) 55.9 ± 22.14	50.0 (18.0–100.0) 53.0 ± 19.69
Parent’s/patient’s global assessment of overall well-being mean ± SD	60.0 (0.0–100.0) 56.7 ± 28.60	67.5 (5.0–100.0) 64.3 ± 25.57	49.5 (0.0–96.0) 47.4 ± 29.79
Number of joints with active arthritis mean ± SD	6.0 (2.0–32.0) 7.7 ± 5.14	6.0 (2.0–22.0) 7.7 ± 4.51	6.0 (2.0–32.0) 7.8 ± 5.89
Number of joints with limitation of movement mean ± SD	4.0 (0.0–17.0) 5.1 ± 4.20	5.0 (0.0–17.0) 5.9 ± 4.68	3.0 (0.0–13.0) 4.2 ± 3.39
CHAQ disability index mean ± SD	0.4 (0.0–2.9) 0.7 ± 0.73	1.0 (0.0–2.9) 0.9 ± 0.75	0.3 (0.0–2.0) 0.4 ± 0.61
Erythrocyte sedimentation rate, mm/h mean ± SD	62.0 (5.0–120.0) 61.0 ± 33.82	64.5 (5.0–114.0) 63.1 ± 31.24	47.0 (6.0–120.0) 58.5 ± 37.14
hsCRP, mg/L mean ± SD	51.0 (4.4–205.6) 59.2 ± 49.41	51.7 (4.4–205.6) 61.1 ± 52.29	41.2 (5.2–143.0) 56.9 ± 46.53

Values are median (range) unless otherwise stated. CHAQ = Childhood Health Assessment Questionnaire; hsCRP = high-sensitivity C-reactive protein; ITT = intention-to-treat; NSAIDs = non-steroidal anti-inflammatory drugs; TCZ = tocilizumab; SD = standard deviation

The median (range) TCZ exposure dose was 6650.5 (138.0–12,762.0) mg, and the median (range) duration of exposure to the study drug was 52.0 (2.0–53.0) weeks.

### Efficacy

#### Primary endpoint

The proportion of patients with a JIA ACR30 response and absence of fever at Week 12 was 87.1% (54/62) (95% CI 78.8%–95.4%) among the overall population, 82.4% (28/34) (95% CI 69.5%–95.2%) in the 12-mg/kg group, and 92.9% (26/28) (95% CI 83.3%–100.0%) in the 8-mg/kg group. The sensitivity analysis results were consistent with those of the primary analysis (Table 2).

**Table 2:** Primary analysis and sensitivity analysis of the primary efficacy endpoint (JIA ACR30 response and absence of fever; observed in patients in the ITT population)

	**TCZ overall population** **N = 62**	**TCZ 12-mg/kg group** **n = 34**	**TCZ 8-mg/kg group** **n = 28**
**Primary analysis (NRI)**			
Week 12			
No. of patients	62	34	28
Responder, n (%)	54 (87.1)	28 (82.4)	26 (92.9)
95% CI	78.8, 95.4	69.5, 95.2	83.3, 100.0
Week 24			
No. of patients	62	34	28
Responder, n (%)	49 (79.0)	25 (73.5)	24 (85.7)
95% CI	68.9, 89.2	58.7, 88.4	72.8, 98.7
Week 52			
No. of patients	62	34	28
Responder, n (%)	54 (87.1)	28 (82.4)	26 (92.9)
95% CI	78.8, 95.4	69.5, 95.2	83.3,100.0
**Sensitivity analysis (Observed)**			
Week 12			
No. of patients	61	33	28
Responder, n (%)	54 (88.5)	28 (84.8)	26 (92.9)
95% CI	80.5, 96.5	72.6, 97.1	83.3, 100.0

ACR = American College of Rheumatology; CI = confidence interval; ITT = intention-to-treat; JIA = juvenile idiopathic arthritis; NRI = non-responder imputation; TCZ = tocilizumab

The mean ± SD values for Week 12 and CFB of the six JIA ACR core components were 20.8 ± 17.71 (CFB − 32.3 ± 18.50) for physician’s global assessment of disease activity, 25.9 ± 22.29 (CFB − 29.1 ± 24.96) for parent’s/patient’s global assessment of overall well-being, 2.4 ± 3.83 (CFB − 5.3 ± 4.40) for number of joints with active arthritis, 1.7 ± 2.79 (CFB − 3.2 ± 3.14) for number of joints with limitation of movement, 3.9 ± 5.72 (CFB − 57.1 ± 34.12) for ESR, and 0.31 ± 0.535 (percent CFB − 57.95% ± 45.583%) for CHAQ disability index.

#### Secondary endpoints

**Fig. 1:**
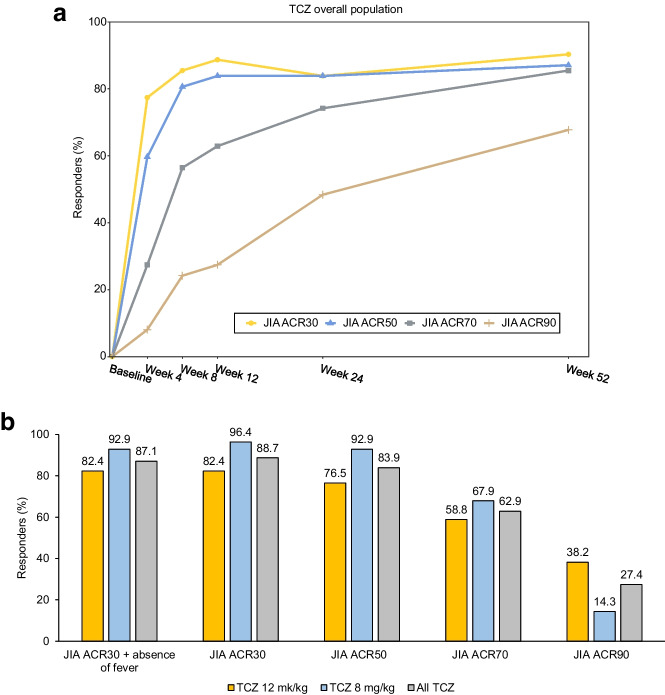
Proportion of JIA ACR 30/50/70/90 responders by week (ITT population) among the TCZ overall population (**A**) and bar chart of the proportion of JIA ACR30 responders with absence of fever and JIA ACR30/50/70/90 responders at Week 12 – non-responders imputation (ITT population) (**B**) ACR = American College of Rheumatology; JIA = juvenile idiopathic arthritis; TCZ = tocilizumab

The proportion of patients with a JIA ACR30 response and absence of fever at Week 52 was 87.1% (54/62) (95% CI 78.8%–95.4%) among the overall population, 82.4% (28/34) (95% CI 69.5%–95.2%) in the 12-mg/kg group, and 92.9% (26/28) (95% CI 83.3%–100.0%) in the 8-mg/kg group (Table 2). The respective proportions of JIA ACR 30/50/70/90 responders were 88.7%, 83.9%, 62.9%, and 27.4% at Week 12; 83.9%, 83.9%, 74.2%, and 48.4% at Week 24; and 90.3%, 87.1%, 85.5%, and 67.7% at Week 52 in the overall population (Fig. 1A and 1B). The proportions of JIA ACR 30/50/70/90 responders by week among those in the 12-mg/kg and 8-mg/kg groups are shown in Fig S2**A and 2B**.

Overall, the proportions of patients with inactive disease were 19.4% (95% CI 9.5%–29.2%) at Week 24 and 35.5% (95% CI 23.6%–47.4%) at Week 52 (Table S1). CHAQ pain scores decreased from baseline, through Weeks 12 and 24, to Week 52, with mean ± SD scores of 48.3 ± 27.15, 18.0 ± 21.60, 10.9 ± 14.69, and 5.5 ± 10.97, respectively (Table 3). At Week 52, the proportion of patients with clinical remission was 24.2% (15/62, 95% CI 13.5%–34.9%) in the overall population, and 23.5% (8/34, 95% CI 9.3%–37.8%) and 25.0% (7/28, 95% CI 9.0%–41.0%) in the 12-mg/kg and 8-mg/kg groups, respectively.

**Table 3:** CHAQ pain scores over time and change from baseline at Weeks 12, 24, and 52

	**TCZ overall population** **N = 62**	**TCZ 12-mg/kg group** **n = 34**	**TCZ 8-mg/kg group** **n = 28**
Baseline	48.3 ± 27.15	55.4 ± 28.21	39.6 ± 23.43
Week 12	18.0 ± 21.60	22.9 ± 26.95	12.9 ± 12.72
CFB	− 29.1 ± 25.61	− 31.3 ± 29.12	− 26.7 ± 21.67
Week 24	10.9 ± 14.69	13.1 ± 17.36	8.5 ± 11.17
CFB	− 36.5 ± 25.59	− 41.1 ± 28.57	− 31.6 ± 21.58
Week 52	5.5 ± 10.97	6.9 ± 11.49	3.9 ± 10.36
CFB	− 41.2 ± 26.54	− 46.7 ± 29.03	− 35.3 ± 22.63

Data are mean ± standard deviation

CHAQ = Childhood Health Assessment Questionnaire; CFB = change from baseline; TCZ = tocilizumab

At Weeks 12, 24, and 52, the mean ± SD hsCRP levels in the overall population were 2.6 ± 10.48 mg/L, 1.1 ± 1.57 mg/L and 5.6 ± 20.69 mg/L, respectively (Fig S3), with corresponding CFB of − 54.3 ± 47.24 mg/L, − 59.1 ± 45.13 mg/L, and − 51.4 ± 46.32 mg/L. Overall, 58 patients had elevated (> ULN) hsCRP level at baseline, among whom 50 (86.2%), 46 (79.3%), and 44 (75.9%) patients had normal (≤ ULN) hsCRP levels at Weeks 12, 24, and 52 (Table S2).

In the overall population, the mean ± SD of corticosteroid dose (prednisone or equivalent) changed from 0.358 ± 0.1623 mg/kg/day at baseline, to 0.293 ± 0.1157 mg/kg/day at Week 12, 0.209 ± 0.1171 mg/kg/day at Week 24, and 0.098 ± 0.1076 mg/kg/day at Week 52 (Fig S4). Most patients used low dose corticosteroids at Week 52. From Week 20 onwards, some patients started to discontinue corticosteroids. The proportion of patients who discontinued corticosteroids was 4.8% (3/62) at Week 24 and 27.4% (17/62) at Week 52.

#### Exploratory endpoints

Mean ± SD CHAQ disability index scores decreased over time from baseline to Week 52 (Table S3). The mean ± SD CFB in CHAQ disability at Week 52 was − 91.20 ± 21.352 in the TCZ overall population, − 85.39 ± 26.869 in the 12-mg/kg group, and − 98.61 ± 5.893 in the 8-mg/kg group. The proportions of observed patients with improvement compared with baseline in the CHAQ disability index were: 52.5% (32/61) at Week 12, 57.9% (33/57) at Week 24, and 61.3% (38/62) at Week 52 (Table S4). The mean ± SD of CHQ physical and psychosocial summary scores increased over time from baseline to Week 52 (Table S5). The parent’s/patient’s global assessment of overall well-being also improved from baseline to Week 52 (Table S6). The mean ± SD CFB in parent’s/patient’s global assessment of overall well-being at Week 52 was − 44.9 ± 27.11 in the TCZ overall population, − 50.7 ± 25.12 in the 12-mg/kg group, and − 36.8 ± 28.21 in the 8-mg/kg group.

The mean ± SD height SDS was − 1.15 ± 1.371 at baseline, and then − 1.20 ± 1.358, − 0.99 ± 1.147, and − 1.04 ± 1.328 at Weeks 12, 24, and 52, respectively. Therefore, at Weeks 12, 24, and 52, the CFB in the mean ± SD height SDS were − 0.07 ± 0.196, − 0.03 ± 0.306, and 0.09 ± 0.416, respectively. The proportions of patients with a minimal clinical improvement (growth rate > 0.25 SDS) in SDS from baseline by Tanner Stage are shown in Table S7.

At baseline, the mean ± SD haemoglobin level was 106.4 ± 13.99 g/L, and over the course of the study the mean ± SD CFB in haemoglobin levels showed increases of 19.8 ± 13.93, 19.1 ± 13.76, and 19.2 ± 16.07 g/L at Weeks 12, 24, and 52, respectively. Regarding platelet counts, the mean ± SD value at baseline was 501.9 ± 151.78 (× 10^9^/L) and this decreased to 283.6 ± 69.64, 278.0 ± 70.46, and 267.4 ± 65.39 (× 10^9^/L) at Weeks 12, 24, and 52, respectively. For leukocyte counts, the baseline mean ± SD value was 14.3 ± 7.57, and this also decreased over time to 7.7 ± 2.74 at Week 12, 7.5 ± 2.74 at Week 24, and 7.3 ± 4.07 (× 10^9^/L) at Week 52. The corresponding CFBs were − 6.7 ± 6.78, − 6.9 ± 7.47, and − 7.0 ± 8.03 (× 10^9^/L) at Weeks 12, 24, and 52, respectively.

### Safety

An overall summary of AEs is shown in Table 4. All 62 patients experienced at least one AE. Treatment-related AEs were reported in 55/62 (88.7%) patients.

**Table 4:** Overall summary of AEs (safety population)

	**TCZ** **overall population** **N = 62**		**TCZ** **12-mg/kg group** **n = 34**		**TCZ** **8-mg/kg group** **n = 28**	
	**Patients** **n (%)**	**No. of events**	**Patients** **n (%)**	**No. of events**	**Patients** **n (%)**	**No. of events**
Any AE	62 (100.0)	585	34 (100.0)	353	28 (100.0)	232
Any treatment-related AE	55 (88.7)	316	31 (91.2)	175	24 (85.7)	141
Any AE leading to treatment interruption	16 (25.8)	35	12 (35.3)	25	4 (14.3)	10
Any AE leading to study discontinuation	4 (6.5)	7	4 (11.8)	7	0	0
Any serious AE	11 (17.7)	21	10 (29.4)	20	1 (3.6)	1
Any treatment-related serious AE	8 (12.9)	18	8 (23.5)	18	0	0
AEs of special interest						
Serious infection	4 (6.5)	4	4 (11.8)	4	0	0
Anaphylactic reaction	2 (3.2)	2	2 (5.9)	2	0	0
Demyelination	1 (1.6)	1	1 (2.9)	1	0	0
Hepatic-related event	2 (3.2)	2	1 (2.9)	1	1 (3.6)	1

AE = adverse event; TCZ = tocilizumab

Most patients experienced Grade < 3 AEs, and 24.2% (15/62) of patients experienced Grade ≥ 3 AEs. Sixteen out of 62 (25.8%) patients experienced AEs leading to treatment interruption including 12/34 (35.3%) patients in the 12-mg/kg group and 4/28 (14.3%) in the 8-mg/kg group. Four out of 34 (6.5%) patients in the 12-mg/kg group and none in the 8-mg/kg group experienced AEs leading to study discontinuation. No death or events of MAS were reported. AEs with an incidence ≥ 20% included upper respiratory tract infection (72.6%, 45 patients), hepatic function abnormal (27.4%, 17 patients), mouth ulceration (22.6%, 14 patients), and pyrexia (21.0%, 13 patients) (Table 5).

**Table 5:** AE (incidence ≥ 20% in any group) by System Organ Class and Preferred Term (safety population)

	**TCZ** **overall population** **N = 62** **n (%)**	**TCZ** **12-mg/kg group** **n = 34** **n (%)**	**TCZ** **8-mg/kg group** **n = 28** **n (%)**
Any AE	62 (100.0)	34 (100.0)	28 (100.0)
Infections and infestations	54 (87.1)	29 (85.3)	25 (89.3)
Upper respiratory tract infection	45 (72.6)	23 (67.6)	22 (78.6)
Paronychia	9 (14.5)	3 (8.8)	6 (21.4)
Blood and lymphatic system disorders	18 (29.0)	14 (41.2)	4 (14.3)
Anaemia	8 (12.9)	7 (20.6)	1 (3.6)
Respiratory, thoracic and mediastinal disorders	19 (30.6)	13 (38.2)	6 (21.4)
Cough	12 (19.4)	9 (26.5)	3 (10.7)
Gastrointestinal disorders	28 (45.2)	16 (47.1)	12 (42.9)
Mouth ulceration	14 (22.6)	6 (17.6)	8 (28.6)
Hepatobiliary disorders	22 (35.5)	8 (23.5)	14 (50.0)
Hepatic function abnormal	17 (27.4)	6 (17.6)	11 (39.3)
General disorders and administration site conditions	15 (24.2)	12 (35.5)	3 (10.7)
Pyrexia	13 (21.0)	10 (29.4)	3 (10.7)
Injury, poisoning and procedural complications	28 (45.2)	18 (52.9)	10 (35.7)
Medication error*	17 (27.4)	12 (35.3)	5 (17.9)

MedDRA version 25.0

AE = adverse event; TCZ = tocilizumab

^*^ Medication error: belonging to special situation

In total, 11/62 (17.7%) of patients experienced SAEs **(**Table S8**)**, including 10/34 (29.4%) patients in the 12-mg/kg group and 1/28 (3.6%) in the 8-mg/kg group (Table 4). All SAEs resolved.

Changes in clinical laboratory tests from baseline to worst post-baseline grade were assessed using CTCAE grade and notable alterations were observed in several parameters. The grading of alkaline phosphatase increased and alanine aminotransferase increased shifted from normal to Grade ≥ 3 in two patients each, while aspartate aminotransferase increased shifted from normal to Grade 4 in two patients. Bilirubin increased shifted from normal to Grade 3 in one patient, creatinine increased shifted from normal to Grade ≥ 3 in two patients, while changes in calcium decreased grading were noted in six patients, with five shifting from normal to Grade 4 and one from Grade 1 to Grade 4. The grading of lymphocyte decreased changed from normal to Grade 3 in one patient whereas neutrophil decreased changed from normal to Grade ≥ 3 in eight patients. A summary of abnormal vital signs is presented in Table S9**.**

## Discussion

The present study is the first multicentre study to evaluate the long-term (52 week) efficacy and safety of treatment with TCZ in Chinese patients with sJIA, and indicates that TCZ has good clinical efficacy in the targeted population. In addition, our results were consistent with those of the global pivotal Phase III study [14], and a real-world observational study in Japan [17], indicating that TCZ has similar efficacy in the Chinese subpopulation and global populations with sJIA.

The JIA ACR30 response with absence of fever, JIA ACR30/50/70/90 response, as well as inactive disease, and clinical remission were shown to steadily increase. The number of joints with active arthritis or limitation of movement continued to decrease throughout the 52-week treatment period. Together, these results indicate effective improvements in systemic symptoms and arthritis.

Levels of hsCRP and ESR rapidly decreased within 4 weeks, and levels of platelets and leukocyte counts tended toward normal values during the treatment period. In addition, haemoglobin levels increased over time, indicating that TCZ quickly improved patients’ inflammatory status and effectively controlled inflammation throughout the study period.

Corticosteroid discontinuation was observed from Week 20 onwards in this study, with consistent reductions in the dosage of oral corticosteroids noted throughout the study period. By Week 52, 27.4% of patients had successfully discontinued corticosteroid use. The Japanese real-world study [17] and the global Phase III study [14] also reported reductions in corticosteroid use, with 12.3% of patients discontinuing corticosteroid use by Week 48 and 52% of patients discontinuing corticosteroid use by Week 52, respectively. The administration of standard TCZ treatment therefore facilitates the tapering or sparing of corticosteroids, which is beneficial for both management of disease and patient treatment outcomes.

The CFB in height SDS demonstrated that height growth exceeds the World Health Organization standard for age at Week 52. This indicates an increase in growth velocity, showing that Chinese patients with sJIA experienced good catch-up growth following treatment with TCZ. Notably, the JIA ACR30 response with absence of fever, along with the JIA ACR30/50/70/90 responses, were observed to be consistently higher in the 8-mg/kg group compared with the 12-mg/kg group. This difference may be due to the higher dosage or concentration of TCZ in the latter.

The exploratory findings showed that treatment with TCZ resulted in continued improvements over time in CHAQ disability index and CHQ physical and psychosocial scores. A consistent improvement in CHAQ disability index scores over time was also shown in the global Phase III study [14]. Although health-related quality of life assessments varied between this study and the real-world study in Japan [17], similar improvements with TCZ treatment were observed.

In this study, treatment-related AEs, SAEs, and AEs leading to study discontinuation were reported in 88.7%, 17.7%, and 6.5% of patients, respectively. Importantly, these results align with those results reported from the global Phase III study (data on file; NCT00642460) and the Japanese study [17], thus demonstrating comparable safety endpoint profiles. More patients developed treatment-related AEs; however, fewer patients developed SAEs. A smaller sample size of the present study may have contributed to this difference between the studies. The most common AE was upper respiratory tract infection (data on file) which is consistent with the results of the global Phase III study [14], while the Japanese real-world study reported infections and infestations as the most common type of AE [17]. In the present study, all SAEs were resolved, and no death or MAS occurred. Therefore, the observed overall safety profile remained benefit–risk balanced.

Overall, the efficacy and safety results of the present study are consistent with those of the global Phase III study [14] and the real-world study in Japan [17]. In patients with sJIA, early treatment with a biological agent such as TCZ and standard therapy to control inflammatory disease and achieve clinical remission is important to avoid MAS or the occurrence of death. The results of the present study suggest that early treatment with TCZ may provide the additional benefits of catch-up growth after treatment with TCZ, rapid discontinuation of corticosteroids, and improved quality of life. The study further provides strong evidence supporting the use of TCZ in patients with sJIA.

The present study has some limitations, such as those inherent to the single-arm open-label design. Although the generalizability of our findings may be limited by the exclusive enrolment of Chinese patients, the global Phase III study accounts for this limitation.

In conclusion, this study reported the long-term (52 week) efficacy and safety of TCZ in Chinese patients with sJIA for the first time. Safety findings in these patients were consistent with the known safety profile of TCZ.

## Supplementary Information

Below is the link to the electronic supplementary material.

Supplementary file1 (DOCX 308 KB)

## Data Availability

For up-to-date details on Roche’s Global Policy on the Sharing of Clinical Information and how to request access to related clinical study documents, see here: https://go.roche.com/data_sharing. Anonymized records for individual patients across more than one data source external to Roche cannot, and should not, be linked due to a potential increase in risk of patient re-identification.
