# Supplementary material for: Efficacy and safety of tocilizumab in Chinese patients with systemic juvenile idiopathic arthritis: a multicentre phase IV trial
Source: Clin Rheumatol. 2024 Sep 16;43(11):3457–67. doi: 10.1007/s10067-024-07126-9 (PMC11489204; doi:10.1007/s10067-024-07126-9)
Supplement: Supplementary file 1 — Supplementary file1 (DOCX 308 KB) [file 10067_2024_7126_MOESM1_ESM.docx]

**Supplemental Materials**

**Supplementary Methods.** List of study sites

1. Beijing Children’s Hospital, Capital Medical University; No. 56 South Lishi Road, Xicheng District, Beijing, China
2. Children’s Hospital of Chongqing Medical University; No. 136 Zhongshan Er Road, Yuzhong District, Chongqing, China
3. Children’s Hospital Capital Institute of Pediatrics; No. 2 Yabao Road, Chaoyang District, Beijing, China
4. Children’s Hospital of Fudan University; No. 399 Wanyuan Road, Shanghai, China
5. Children’s Hospital, Zhejiang University School of Medicine; No. 3333 Binsheng Road, Hangzhou, China
6. Shanghai Children’s Medical Center, Shanghai Jiaotong University School of Medicine; No. 1678, Dongfang Road, Pudong New District, Shanghai, China
7. The First Hospital of Jilin University; No. 1, Xinmin Street, Chaoyang District, Changchun, Jilin, China
8. The Second Affiliated Hospital and Yuying Children’s Hospital of Wenzhou Medical University; No. 109, Xueyuan West Road, Lucheng District, Wenzhou, Zhejiang, China
9. Children’s Hospital of Nanjing Medical University; No. 72, Guangzhou Road, Gulou District, Nanjing, China
10. Sun Yat-sen Memorial Hospital, Sun Yat-sen University; No.107, Yanjiang West Road, Yuexiu District, Guangzhou, China

**Supplementary Figure S1.** Patient disposition

ITT = intention-to-treat; TCZ = tocilizumab.

**
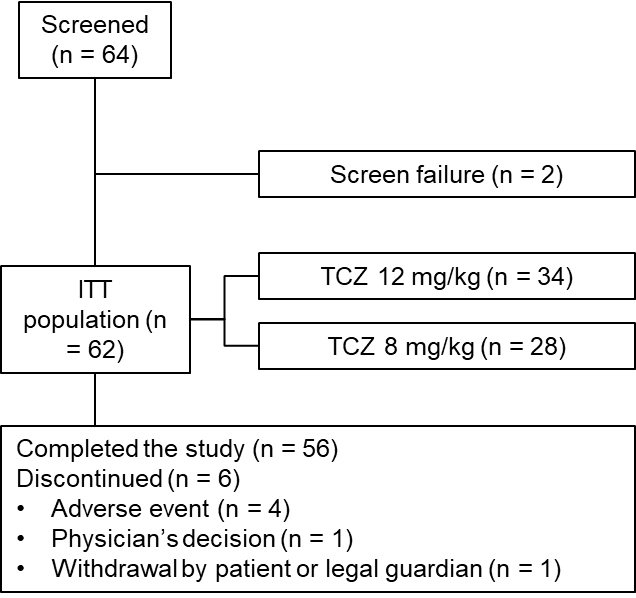
**

**Supplementary Figure S2.** Proportion of JIA ACR 30/50/70/90 responders by week (intention-to-treat population) among patients in the (**A**) 12- and (**B**) 8-mg/kg groups

ACR = American College of Rheumatology; JIA = juvenile idiopathic arthritis; TCZ = tocilizumab.

**Supplementary Figure S2A
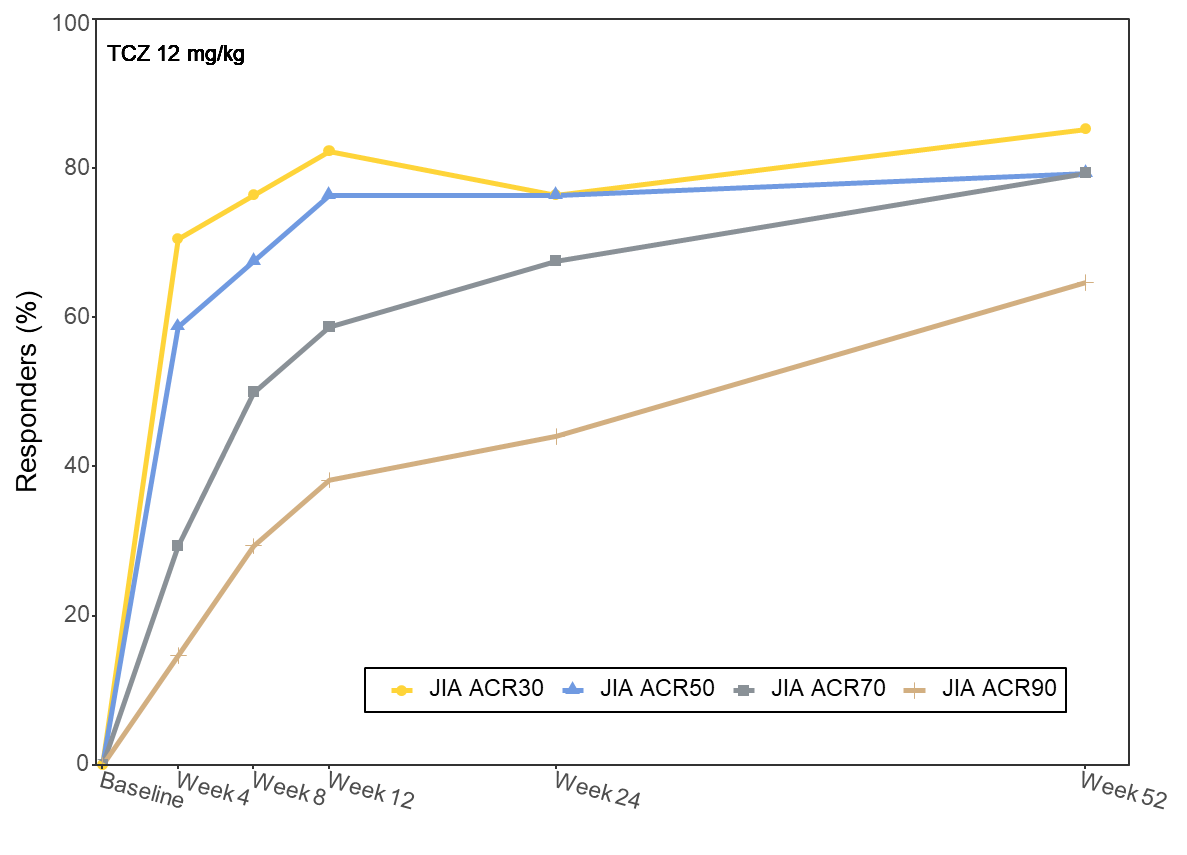
**

**Figure S2B
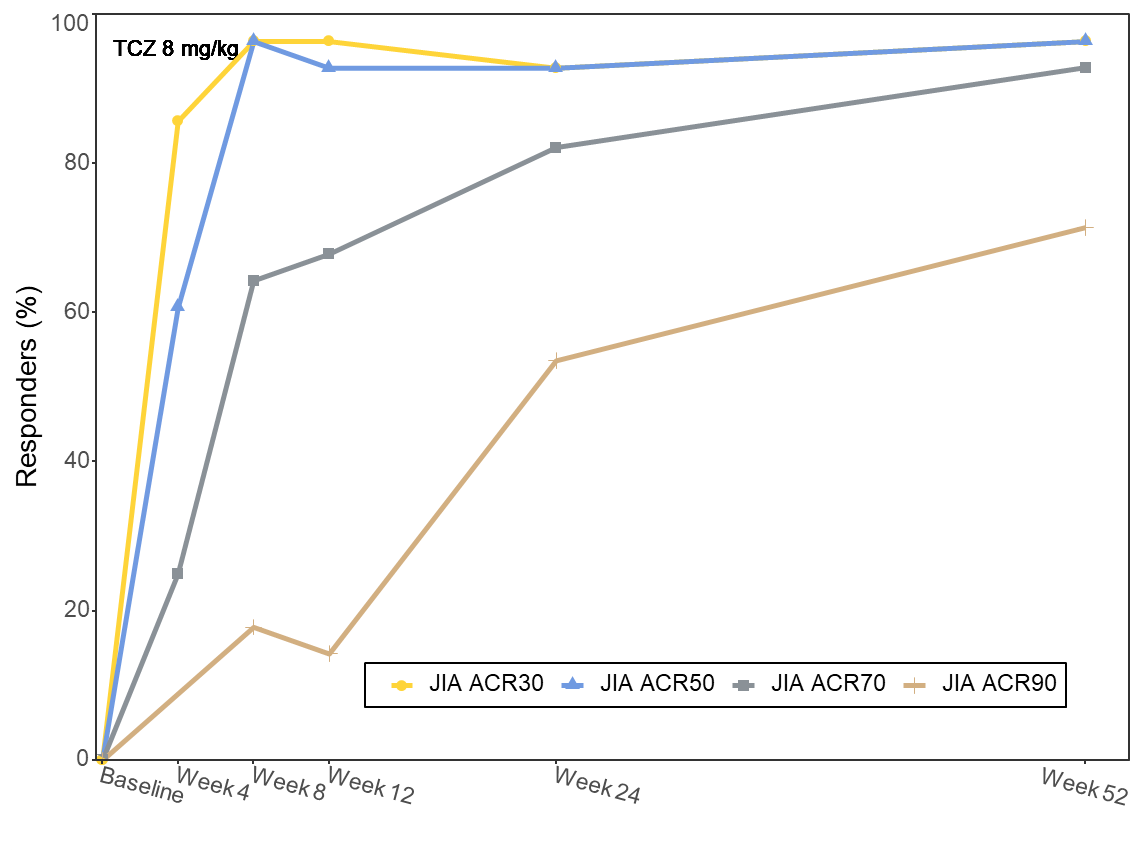
**

**Supplementary Figure S3.** Mean hsCRP levels over time (ITT population)

hsCRP = high-sensitivity C-reactive protein; SE = standard error; TCZ = tocilizumab

**
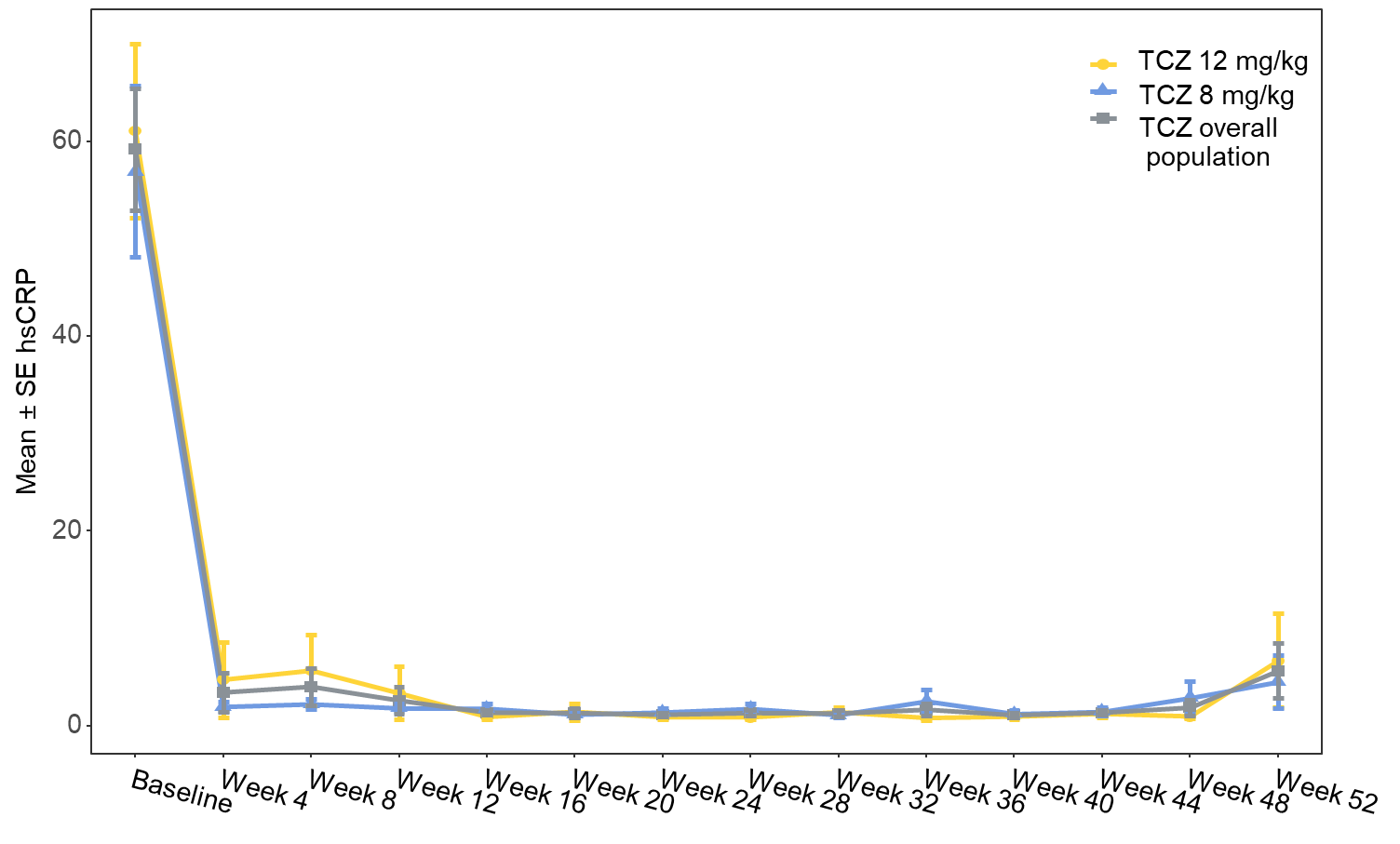
**

**Supplementary Figure S4.** Mean corticosteroid dose over time (ITT population)

ITT = intention-to-treat; SE = standard error; TCZ = tocilizumab.


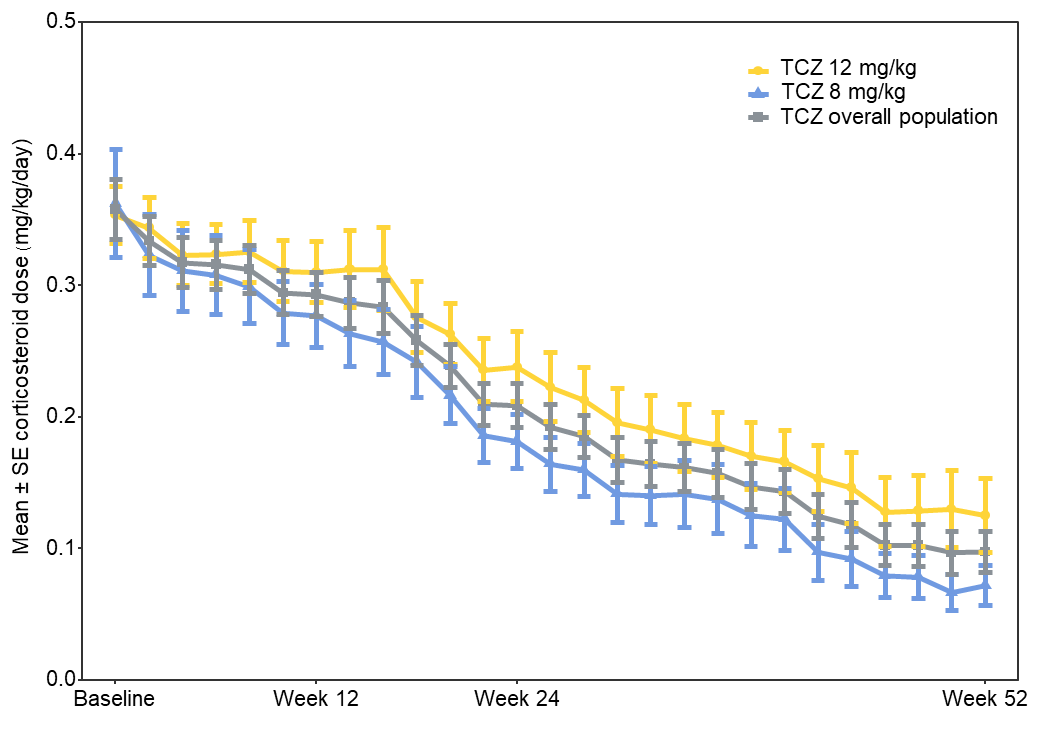


**Supplementary Table S1.** Patients with inactive disease at Weeks 12, 24, and 52 (ITT population)

|  | **TCZ overall population**  **N = 62** | **TCZ 12-mg/kg group**  **n = 34** | **TCZ 8-mg/kg group**  **n = 28** |
| --- | --- | --- | --- |
| Week 12 |  |  |  |
| Inactive disease, n (%) | 10 (16.1) | 6 (17.6) | 4 (14.3) |
| 95% CI | 7.0, 25.3 | 4.8, 30.5 | 1.3, 27.2 |
| Week 24 |  |  |  |
| Inactive disease, n (%) | 12 (19.4) | 8 (23.5) | 4 (14.3) |
| 95% CI | 9.5, 29.2 | 9.3, 37.8 | 1.3, 27.2 |
| Week 52 |  |  |  |
| Inactive disease, n (%) | 22 (35.5) | 13 (38.2) | 9 (32.1) |
| 95% CI | 23.6, 47.4 | 21.9, 54.6 | 14.8, 49.4 |

CI = confidence interval; ITT = intention-to-treat; TCZ = tocilizumab.

**Supplementary** **Table S2.** Proportions of patients who had elevated hsCRP levels at baseline and normal hsCRP levels at Weeks 12, 24, and 52 (ITT population)

|  | **TCZ overall population**  **N = 62** | **TCZ 12-mg/kg group**  **n = 34** | **TCZ 8-mg/kg group**  **n = 28** |
| --- | --- | --- | --- |
| Week 12 |  |  |  |
| No. of patients | 58 | 32 | 26 |
| Normal (≤ULN) hsCRP, n (%) | 50 (86.2) | 26 (81.3) | 24 (92.3) |
| 95% CI | 77.3, 95.1 | 67.7, 94.8 | 82.1, 100.0 |
| Week 24 |  |  |  |
| No. of patients | 58 | 32 | 26 |
| Normal (≤ULN) hsCRP, n (%) | 46 (79.3) | 24 (75.0) | 22 (84.6) |
| 95% CI | 68.9, 89.7 | 60.0, 90.0 | 70.7, 98.5 |
| Week 52 |  |  |  |
| No. of patients | 58 | 32 | 26 |
| Normal (≤ULN) hsCRP, n (%) | 44 (75.9) | 24 (75.0) | 20 (76.9) |
| 95% CI | 64.8, 86.9 | 60.0, 90.0 | 60.7, 93.1 |

CI = confidence interval; hsCRP = high-sensitivity C-reactive protein; ITT = intention-to-treat; TCZ = tocilizumab; ULN = upper limit of normal.

**Supplementary Table S3.** Mean score and percentage change from baseline in CHAQ disability index score by week (ITT population)

|  | **Score** | | | | | | **Change from baseline (%)** | | | | | |
| --- | --- | --- | --- | --- | --- | --- | --- | --- | --- | --- | --- | --- |
|  | **n** | **Mean** | **SD** | **Median** | **Min** | **Max** | **n** | **Mean** | **SD** | **Median** | **Min** | **Max** |
| **TCZ overall population**  **(N = 62)** |  |  |  |  |  |  |  |  |  |  |  |  |
| Baseline | 62 | 0.71 | 0.727 | 0.40 | 0.00 | 2.86 |  |  |  |  |  |  |
| Week 12 | 57 | 0.31 | 0.535 | 0.00 | 0.00 | 2.50 | 42 | −57.95 | 45.583 | −73.30 | −100.00 | 56.25 |
| Week 24 | 53 | 0.22 | 0.484 | 0.00 | 0.00 | 2.60 | 38 | −72.19 | 39.532 | −96.75 | −100.00 | 62.50 |
| Week 52 | 56 | 0.11 | 0.310 | 0.00 | 0.00 | 1.38 | 41 | −91.20 | 21.352 | −100.00 | −100.00 | −8.00 |
| **TCZ 12-mg/kg group**  **(N = 34)** |  |  |  |  |  |  |  |  |  |  |  |  |
| Baseline | 34 | 0.93 | 0.746 | 1.00 | 0.00 | 2.86 |  |  |  |  |  |  |
| Week 12 | 29 | 0.43 | 0.618 | 0.25 | 0.00 | 2.50 | 23 | −46.42 | 51.211 | −49.71 | −100.00 | 56.25 |
| Week 24 | 27 | 0.33 | 0.605 | 0.00 | 0.00 | 2.60 | 21 | −65.51 | 42.501 | −78.29 | −100.00 | 62.50 |
| Week 52 | 29 | 0.21 | 0.411 | 0.00 | 0.00 | 1.38 | 23 | −85.39 | 26.869 | −100.00 | −100.00 | −8.00 |
| **TCZ 8-mg/kg group**  **(N = 28)** |  |  |  |  |  |  |  |  |  |  |  |  |
| Baseline | 28 | 0.44 | 0.613 | 0.30 | 0.00 | 2.00 |  |  |  |  |  |  |
| Week 12 | 28 | 0.19 | 0.407 | 0.00 | 0.00 | 1.75 | 19 | −71.90 | 33.945 | −79.79 | −100.00 | 0.00 |
| Week 24 | 26 | 0.10 | 0.279 | 0.00 | 0.00 | 1.38 | 17 | −80.45 | 35.001 | −100.00 | −100.00 | 0.00 |
| Week 52 | 27 | 0.01 | 0.027 | 0.00 | 0.00 | 0.10 | 18 | −98.61 | 5.893 | −100.00 | −100.00 | −75.00 |

CHAQ = Childhood Health Assessment Questionnaire; ITT = intention-to-treat; SD = standard deviation; TCZ = tocilizumab.

**Supplementary Table S4.** Proportions of patients with improvement compared with baseline in the CHAQ disability index

|  | **TCZ overall population**  **N = 62** | **TCZ 12–mg/kg group**  **n = 34** | **TCZ 8-mg/kg group**  **n = 28** |
| --- | --- | --- | --- |
| Week 12 | 61 | 33 | 28 |
| n (%) | 32 (52.5) | 16 (48.5) | 16 (57.1) |
| 95% CI | 39.9, 65.0 | 31.4, 65.5 | 38.8, 75.5 |
| Week 24 |  |  |  |
| n (%) | 33 (57.9) | 19 (61.3) | 14 (53.8) |
| 95% CI | 45.1, 70.7 | 44.1, 78.4 | 34.7, 73.0 |
| Week 52 |  |  |  |
| n (%) | 38 (61.3) | 22 (64.7) | 16 (57.1) |
| 95% CI | 49.2, 73.4 | 48.6, 80.8 | 38.8, 75.5 |

95% CI is from two-sided Wald confidence limits.

CHAQ disability index improvement is defined as change from baseline ≤−0.13.

CHAQ = Childhood Health Assessment Questionnaire; CI = confidence interval; TCZ = tocilizumab.

**Supplementary Table S5.** Mean score and change from baseline in CHQ physical and psychosocial summary scores by week (ITT population)

|  | **Actual value** | | | | | | **Change from baseline** | | | | | |
| --- | --- | --- | --- | --- | --- | --- | --- | --- | --- | --- | --- | --- |
| **CHQ physical summary scores** | **n** | **Mean** | **SD** | **Median** | **Min** | **Max** | **n** | **Mean** | **SD** | **Median** | **Min** | **Max** |
| **TCZ overall population**  **(N = 62)** |  |  |  |  |  |  |  |  |  |  |  |  |
| Baseline | 62 | 23.4 | 16.00 | 23.9 | −5.1 | 53.4 |  |  |  |  |  |  |
| Week 12 | 58 | 40.0 | 11.81 | 41.8 | 6.1 | 57.0 | 58 | 15.5 | 13.98 | 14.8 | −12.8 | 47.7 |
| Week 24 | 49 | 41.4 | 11.40 | 44.0 | −1.9 | 57.5 | 49 | 17.8 | 14.55 | 17.4 | −17.7 | 44.8 |
| Week 52 | 56 | 44.4 | 9.37 | 46.0 | 10.8 | 57.9 | 56 | 19.6 | 16.37 | 17.9 | −15.7 | 56.1 |
| **TCZ 12-mg/kg group**  **(n = 34)** |  |  |  |  |  |  |  |  |  |  |  |  |
| Baseline | 34 | 18.9 | 15.17 | 15.1 | −5.1 | 48.1 |  |  |  |  |  |  |
| Week 12 | 30 | 39.3 | 13.20 | 41.3 | 6.1 | 57.0 | 30 | 19.0 | 15.72 | 17.9 | −4.3 | 47.7 |
| Week 24 | 26 | 40.5 | 12.92 | 42.8 | −1.9 | 56.8 | 26 | 20.7 | 16.72 | 25.6 | −17.7 | 44.8 |
| Week 52 | 29 | 43.1 | 11.39 | 46.6 | 10.8 | 55.9 | 29 | 22.2 | 17.82 | 28.4 | −15.7 | 56.1 |
| **TCZ 8-mg/kg group**  **(n = 28)** |  |  |  |  |  |  |  |  |  |  |  |  |
| Baseline | 28 | 28.9 | 15.51 | 28.6 | –3.2 | 53.4 |  |  |  |  |  |  |
| Week 12 | 28 | 40.7 | 10.29 | 42.2 | 12.1 | 55.8 | 28 | 11.8 | 10.95 | 13.8 | −12.8 | 30.9 |
| Week 24 | 23 | 42.5 | 9.59 | 45.1 | 19.2 | 57.5 | 23 | 14.5 | 11.09 | 16.4 | −8.3 | 35.0 |
| Week 52 | 27 | 45.7 | 6.49 | 44.4 | 34.1 | 57.9 | 27 | 16.7 | 14.45 | 17.4 | −4.9 | 42.7 |
| **CHQ psychosocial summary scores** |  |  |  |  |  |  |  |  |  |  |  |  |
| **TCZ overall population**  **(N = 62)** |  |  |  |  |  |  |  |  |  |  |  |  |
| Baseline | 62 | 41.3 | 11.08 | 42.7 | 19.0 | 63.6 |  |  |  |  |  |  |
| Week 12 | 58 | 47.0 | 10.10 | 49.9 | 24.1 | 61.2 | 58 | 4.9 | 8.76 | 4.8 | −16.7 | 29.1 |
| Week 24 | 49 | 47.2 | 10.69 | 49.2 | 16.4 | 64.3 | 49 | 4.7 | 10.14 | 4.6 | −23.4 | 24.1 |
| Week 52 | 56 | 50.8 | 8.21 | 51.6 | 31.4 | 63.6 | 56 | 8.5 | 10.43 | 10.0 | −20.4 | 31.8 |
| **TCZ 12-mg/kg group**  **(n = 34)** |  |  |  |  |  |  |  |  |  |  |  |  |
| Baseline | 34 | 41.3 | 11.16 | 41.3 | 19.0 | 60.3 |  |  |  |  |  |  |
| Week 12 | 30 | 46.6 | 10.29 | 49.7 | 27.1 | 61.0 | 30 | 3.9 | 8.82 | 3.9 | −16.7 | 26.3 |
| Week 24 | 26 | 46.6 | 11.90 | 47.2 | 16.4 | 64.3 | 26 | 2.8 | 10.35 | 2.7 | −23.4 | 24.1 |
| Week 52 | 29 | 50.6 | 7.86 | 51.2 | 37.3 | 63.6 | 29 | 7.7 | 10.79 | 9.3 | −20.4 | 29.5 |
| **TCZ 8-mg/kg group**  **(n = 28)** |  |  |  |  |  |  |  |  |  |  |  |  |
| Baseline | 28 | 41.3 | 11.19 | 43.1 | 20.5 | 63.6 |  |  |  |  |  |  |
| Week 12 | 28 | 47.4 | 10.06 | 50.4 | 24.1 | 61.2 | 28 | 6.1 | 8.71 | 6.9 | −10.6 | 29.1 |
| Week 24 | 23 | 48.0 | 9.33 | 49.8 | 24.5 | 57.9 | 23 | 6.9 | 9.64 | 6.7 | −11.8 | 23.4 |
| Week 52 | 27 | 51.0 | 8.71 | 52.0 | 31.4 | 62.3 | 27 | 9.3 | 10.18 | 11.0 | −7.2 | 31.8 |

CHQ = Child Health Questionnaire; ITT = intention-to-treat; SD = standard deviation; TCZ = tocilizumab.

**Supplementary Table S6.** Mean score and change from baseline in parent’s/patient’s global assessment of overall well-being by week (ITT population)

|  | **Score** | | | | | | **Change from baseline** | | | | | |
| --- | --- | --- | --- | --- | --- | --- | --- | --- | --- | --- | --- | --- |
| **Parent’s/patient’s global assessment of overall well-being** | **n** | **Mean** | **SD** | **Median** | **Min** | **Max** | **n** | **Mean** | **SD** | **Median** | **Min** | **Max** |
| **TCZ overall population**  **(N = 62)** |  |  |  |  |  |  |  |  |  |  |  |  |
| Baseline | 62 | 56.7 | 28.60 | 60.0 | 0.0 | 100.0 |  |  |  |  |  |  |
| Week 12 | 57 | 25.9 | 22.29 | 20.0 | 0.0 | 100.0 | 57 | −29.1 | 24.96 | −30.0 | −88.0 | 10.0 |
| Week 24 | 53 | 14.2 | 15.44 | 10.0 | 0.0 | 80.0 | 53 | −39.5 | 26.26 | −40.0 | −91.0 | 13.0 |
| Week 52 | 56 | 9.3 | 13.66 | 5.0 | 0.0 | 59.0 | 56 | −44.9 | 27.11 | −47.5 | −94.0 | 0.0 |
| **TCZ 12-mg/kg group**  **(n = 34)** |  |  |  |  |  |  |  |  |  |  |  |  |
| Baseline | 34 | 64.3 | 25.57 | 67.5 | 5.0 | 100.0 |  |  |  |  |  |  |
| Week 12 | 29 | 28.9 | 25.28 | 20.0 | 0.0 | 100.0 | 29 | −33.4 | 24.10 | −30.0 | −88.0 | 7.0 |
| Week 24 | 27 | 17.8 | 17.99 | 15.0 | 0.0 | 80.0 | 27 | −43.8 | 26.84 | −43.0 | −91.0 | 13.0 |
| Week 52 | 29 | 11.0 | 15.55 | 5.0 | 0.0 | 59.0 | 29 | −50.7 | 25.12 | −54.0 | −93.0 | –5.0 |
| **TCZ 8-mg/kg group**  **(n = 28)** |  |  |  |  |  |  |  |  |  |  |  |  |
| Baseline | 28 | 47.4 | 29.79 | 49.5 | 0.0 | 96.0 |  |  |  |  |  |  |
| Week 12 | 28 | 22.8 | 18.66 | 20.0 | 0.0 | 80.0 | 28 | −24.6 | 25.49 | −20.5 | −71.0 | 10.0 |
| Week 24 | 26 | 10.4 | 11.40 | 6.0 | 0.0 | 38.0 | 26 | −35.1 | 25.41 | −31.5 | −85.0 | 3.0 |

ITT = intention-to-treat; SD = standard deviation; TCZ = tocilizumab.

**Supplementary Table S7.** Proportions of patients with minimal clinical improvement (growth rate >0.25 SDS) in height SDS from baseline by Tanner Stage (ITT population, observed)

|  | **TCZ overall population**  **N = 44** | **TCZ 12-mg/kg group**  **n = 32** | **TCZ 8-mg/kg group**  **n = 12** |
| --- | --- | --- | --- |
| **Tanner Stage 1** |  |  |  |
| Week 12 | 40 | 28 | 12 |
| n (%) | 2 (5.0) | 2 (7.1) | 0 |
| 95% CI | 0.0, 11.8 | 0.0, 16.7 | 0.0, 0.0 |
| Week 24 | 36 | 26 | 10 |
| n (%) | 7 (19.4) | 5 (19.2) | 2 (20.0) |
| 95% CI | 6.5, 32.4 | 4.1, 34.4 | 0.0, 44.8 |
| Week 52 | 40 | 32 | 8 |
| n (%) | 13 (32.5) | 10 (31.3) | 3 (37.5) |
| 95% CI | 18.0, 47.0 | 15.2, 47.3 | 4.0, 71.0 |
| **Tanner Stage 2** |  |  |  |
| Week 24 | 6 | 0 | 6 |
| n (%) | 1 (16.7) | 0 | 1 (16.7) |
| 95% CI | 0.0, 46.5 | –, – | 0.0, 46.5 |
| **Tanner Stage 3** |  |  |  |
| Week 24 | 4 | 0 | 4 |
| n (%) | 1 (25.0) | 0 | 1 (25.0) |
| 95% CI | 0.0, 67.4 | –, – | 0.0, 67.4 |
| Week 52 | 6 | 0 | 6 |
| n (%) | 2 (33.3) | 0 | 2 (33.3) |
| 95% CI | 0.0, 71.1 | –, – | 0.0, 71.1 |
| **Tanner Stage 4** |  |  |  |
| Week 12 | 4 | 0 | 4 |
| n (%) | 1 (25.0) | 0 | 1 (25.0) |
| 95% CI | 0.0, 67.4 | –, – | 0.0, 67.4 |
| Week 24 | 4 | 0 | 4 |
| n (%) | 1 (25.0) | 0 | 1 (25.0) |
| 95% CI | 0.0, 67.4 | –, – | 0.0, 67.4 |

CI = confidence interval; ITT = intention-to-treat; SDS = standard deviation score; TCZ = tocilizumab.

**Supplementary Table S8.** Summary of serious AEs by system organ class and preferred term (safety population)

|  | **TCZ overall population**  **N = 62 (%)** | **TCZ**  **12-mg/kg group**  **n = 34 (%)** | **TCZ**  **8-mg/kg group**  **n = 28 (%)** |
| --- | --- | --- | --- |
| Any serious AE | 11 (17.7) | 10 (29.4) | 1 (3.6) |
| Infections and infestations | 4 (6.5) | 4 (11.8) | 0 |
| Bronchitis | 2 (3.2) | 2 (5.9) | 0 |
| Infection | 1 (1.6) | 1 (2.9) | 0 |
| Pneumonia | 1 (1.6) | 1 (2.9) | 0 |
| Blood and lymphatic system disorders | 2 (3.2) | 2 (5.9) | 0 |
| Neutropenia | 2 (3.2) | 2 (5.9) | 0 |
| Immune system disorders | 1 (1.6) | 1 (2.9) | 0 |
| Seasonal allergy | 1 (1.6) | 1 (2.9) | 0 |
| Psychiatric disorders | 1 (1.6) | 1 (2.9) | 0 |
| Apathy | 1 (1.6) | 1 (2.9) | 0 |
| Nervous system disorders | 1 (1.6) | 1 (2.9) | 0 |
| Leukoencephalopathy | 1 (1.6) | 1 (2.9) | 0 |
| Gastrointestinal disorders | 1 (1.6) | 1 (2.9) | 0 |
| Vomiting | 1 (1.6) | 1 (2.9) | 0 |
| Hepatobiliary disorders | 1 (1.6) | 1 (2.9) | 0 |
| Hepatic function abnormal | 2 (3.2) | 1 (2.9) | 0 |
| Liver injury | 1 (1.6) | 1 (2.9) | 0 |
| General disorders and administration site conditions | 1 (1.6) | 0 | 1 (3.6) |
| Pyrexia | 2 (3.2) | 2 (5.9) | 0 |
| Chills | 2 (3.2) | 2 (5.9) | 0 |
| Injury, poisoning and procedural complications | 1 (1.6) | 1 (2.9) | 0 |
| Fracture | 1 (1.6) | 1 (2.9) | 0 |

MedDRA version 25.0.

AE = adverse event; TCZ = tocilizumab.

**Supplementary Table S9.** Summary of abnormal vital signs (safety population)

|  | **TCZ overall population**  **N = 62 (%)** | **TCZ**  **12-mg/kg group**  **n = 34 (%)** | **TCZ**  **8-mg/kg group**  **n = 28 (%)** |
| --- | --- | --- | --- |
| Diastolic blood pressure, mmHg |  |  |  |
| Value <LLN and CFB <–20 | 16 (25.8) | 7 (20.6) | 9 (32.1) |
| Value >ULN and CFB >20 | 9 (14.5) | 6 (17.6) | 3 (10.7) |
| Systolic blood pressure, mmHg |  |  |  |
| Value <LLN and CFB <–20 | 14 (22.6) | 6 (17.6) | 8 (28.6) |
| Value >ULN and CFB >20 | 7 (11.3) | 1 (2.9) | 6 (21.4) |
| Pulse rate, beats/min |  |  |  |
| Value <LLN and CFB <–20 | 10 (16.1) | 7 (20.6) | 3 (10.7) |
| Value >ULN and CFB >20 | 9 (14.5) | 4 (11.8) | 5 (17.9) |
| Weight, kg |  |  |  |
| Percent CFB ≥10 | 44 (71.0) | 28 (82.4) | 16 (57.1) |
| Percent CFB <–10 | 2 (3.2) | 0 | 2 (7.1) |

CFB = change from baseline; LLN = lower limit of normal; TCZ = tocilizumab; ULN = upper limit of normal.
